# Editorial: Focus feature on consciousness and cognition

**DOI:** 10.1162/netn_e_00273

**Published:** 2022-10-01

**Authors:** Randy McIntosh, Sean Hill, Olaf Sporns

**Affiliations:** Institute for Neuroscience and Neurotechnology, Department of Biomedical Physiology and Kinesiology, Simon Fraser University, Burnaby, BC, Canada; Krembil Centre for Neuroinformatics, Departments of Psychiatry and Psychology, University of Toronto, Toronto, ON, Canada; Department of Psychological and Brain Sciences, Indiana University, Bloomington, IN, USA

**Keywords:** Connectivity, Brain networks, Brain dynamics, Consciousness, Cognition

## Abstract

Consciousness and cognition are an increasing focus of theoretical and experimental research in neuroscience, leveraging the methods and tools of brain dynamics and connectivity. This Focus Feature brings together a collection of articles that examine the various roles of brain networks in computational and dynamic models, and in studies of physiological and neuroimaging processes that underpin and enable behavioral and cognitive function.

Network neuroscience is a relatively new field but has arguably transformed how we approach the study of the brain. Over the past 20 years, the focus on connections and circuits evolved in parallel with technological developments that gave us the ability to measure much of the neural activity unfolding in the functioning brain. These new data brought challenges to our thinking and gave rise to new ideas and concepts—on dynamics and networks, and on the roles of connectivity and information in shaping cognition and behavior. Many of these ideas arose and challenges were addressed openly in a series of meetings called the Brain Connectivity Workshop (BCW). The inaugural event took place in Dusseldorf, Germany, in 2002, organized by Rolf Kotter and Karl Friston. The aim was to establish a dialogue between researchers in theoretical and computational neuroscience, and in neuroscience methods and experimentation centering on the emerging field of brain connectivity and networks. The meeting format was and still is unique, with an emphasis on a brief presentation (no more than 15 min) and extended periods of discussion to focus on the core issues of brain function. The 19th BCW meeting, originally planned to take place in Toronto but cancelled due to Covid-19, happened virtually in 2021. It was organized to bring some perspective on how the field of network neuroscience had evolved, with a particular focus on whether the network perspective had supported any new ideas about higher brain function. Arguably, the answer to this was a cautious “yes.”

After the meeting, we invited the attendees to submit papers that captured some of their key messages. What is common for all these papers is the use of network terms and concepts to make their case. The clearest examples of this are the papers from Liu, Betzel, and Misic (2022) and Hilgetag, Goulas, and Changeux (2022). Liu et al. examine the link between functional and structural connectivity, where functional connections are reweighted based on their underlying structural and geometric embedding. They provide a quantitative framework for evaluating polysynaptic functional connections. Hilgetag et al. provide a complementary paper that links the connectivity features of the human brain to our unique cognitive abilities. These features span both local and global aspects of brain network organization. The importance of the temporal domains is central to the papers from John et al. (2022) and Wang and Halassa (2022). John et al. emphasize time and propose dynamical systems theory as a framework to evolve a better understanding of the temporal richness of network dynamics. Wang and Halassa present a detailed exploration of thalamocortical interactions in learning, showing that cortex-thalamus cortico-striatal loops interact over different timescales to enable meta-learning. Network dynamics are more directly related to cognitive functions and extended to clinical applications in the remaining papers. Demertzi et al. (2022) explore the patterns of interactions between networks across time in relation to segregation and integration. They link anticorrelations to variations in local and global network inhibition, which is purported to be essential for conscious mental activity. Katsumi, Theriault, Quigley, and Barrett (2022) connect allostatic prediction (predictions needed to coordinate internal systems) and network gradients of functional connectivity. This work moves associations of psychological functions from specific regions or networks to whole-brain phenomena with allostatic features. Kang, Galdo, and Turner (2022) further expand the structure-function relation by estimating clusters of regions based on structural connectivity, and then use the clusters as constraints in a factor analysis of functional connectivity and map their relation to a range of cognitive functions. Karvelis et al. (2022) review studies that use computational models based on empirical data to predict treatment response in major depressive disorder. They suggest that a generative modeling approach may be more sensitive for prediction but also make the case that treatment outcomes should be evaluated across a continuum and with multiple measures to better capture a patient’s unique circumstance. Our Focus Feature concludes with work from Cruzat et al. (2022), which examines changes in signatures of brain network dynamics (turbulence) in relation to the administration of psychedelic drugs. One common finding across drug types was that the psychedelic state seems to increase information transfer through long-range spatial scales. The thread of network-level explanations and predictions is common across all papers and illustrates the advances in neuroscience that have originated from the network perspective.

Finally, if you want to more directly experience the talks and discussions that took place at the meeting, you can access all of them on the BCW YouTube channel. We feel that the unique format of the Brain Connectivity Workshop, which prioritizes intensive audience participation and interaction, is something we desperately need in present-day neuroscience. We tend to get overwhelmed by technological wizardry and the accompanying abundance of data while losing sight of what we are trying to learn. BCW gives us a chance to talk about this. We hope you find inspiration in these pages.
